# The Association between Adiposity and the Risk of Glaucoma: A Meta-Analysis

**DOI:** 10.1155/2017/9787450

**Published:** 2017-06-12

**Authors:** Weiming Liu, Jiawen Ling, Yiyi Chen, Yan Wu, Peirong Lu

**Affiliations:** ^1^Department of Ophthalmology, The First Affiliated Hospital of Soochow University, 188 Shizi Street, Suzhou 215006, China; ^2^Department of Ophthalmology, The Third People's Hospital of Zhangjiagang, Zhangjiagang, China

## Abstract

**Purpose:**

This meta-analysis was conducted to determine the potential association between adiposity and glaucoma incidence.

**Materials and Methods:**

A comprehensive literature search was performed in PubMed and ISI Web of Science. A meta-analysis was conducted using STATA software.

**Results:**

Fifteen eligible studies involving 2,445,980 individuals were included to investigate the association between adiposity and glaucoma incidence. The relative risks (RRs) were pooled with 95% confidence intervals (CI) by using a random-effects model. The pooled RR between adiposity and elevated intraocular pressure (IOP) was 1.73 (95% CI, 1.18–2.54), whereas that between adiposity and open-angle glaucoma (OAG) was 0.97 (95% CI, 0.83–1.13). The pooled RR between abdominal adiposity and glaucoma was 1.28 (95% CI, 1.15–1.41), whereas that between general adiposity and glaucoma was 1.09 (95% CI, 0.87–1.37). Results of subgroup analysis by sex indicated the association between adiposity and glaucoma in the female group (RR, 1.31; 95% CI, 1.05–1.64), but not in the male group (RR, 1.11; 95% CI, 0.77–1.60). The pooled RR of cohort studies and cross-sectional studies were 1.00 (95% CI, 0.84–1.20) and 1.22 (95% CI, 0.89–1.66), respectively.

**Conclusions:**

Adiposity has a higher risk of elevated IOP, and abdominal adiposity has a positive association with glaucoma, especially in female patients.

## 1. Introduction

Glaucoma is the first leading cause of irreversible blindness [1]. OAG is the most common type of glaucoma [1]. The main risk factor for OAG is considered to be elevated IOP [2] and other systematic risk factors, including older age, family history of OAG [2], diabetes mellitus [3], alcohol consumption [4], hypertension, and cigarette smoking [5].

Globally, being overweight and adiposity pose a threat to children and adolescents both in developed and developing countries. However, whether anthropometric factors, such as general adiposity or abdominal adiposity, are determinants of OAG or elevated IOP risk is still unclear.

To assess body-weight status, body mass index (BMI) is usually used as an indicator of general adiposity, and waist circumference (WC) or waist-to-hip ratio (WHR) is used for abdominal adiposity [6]. Previous studies [7, 8] have found that a higher BMI is related to glaucoma incidence, while some studies [2, 9] did not report a positive association between BMI and elevated IOP or OAG. Other studies have shown positive trends and significant correlations between higher WC or WHR and elevated IOP [10, 11]. These inconsistent results prompted this meta-analysis to provide a more accurate estimate of the association between adiposity and glaucoma incidence.

## 2. Materials and Methods

### 2.1. Search Strategy

This meta-analysis was conducted under the guidance of PRISMA [12]. A systematic search was carried out in PubMed and ISI Web of Science before December 2016, using the following terms: ((metabolic syndrome) OR (overweight) OR (obesity) OR (adiposity) OR (body mass index) OR (BMI) OR (intra-abdominal fat) OR (waist hip ratio) OR (waist circumference) OR (Anthropometric)) AND ((glaucoma) OR (intraocular pressure) OR (ocular hypertension) OR (open-angle glaucoma) OR (normal tension glaucoma) OR (high tension glaucoma)). The search was run according to Medical Subject Headings (MeSH), without restriction to regions, or publication types. The language was restricted to English. Citations for related articles were detected for additional publications. Where several reports related to the same study, only the most recent report was used.

### 2.2. Inclusion and Exclusion Criteria

The published study was included in this meta-analysis if it met all the following criteria: (1) reports the association of adiposity, BMI, WC, or WHR with glaucoma, or elevated IOP; (2) adopts a cohort, case-control or cross-sectional design; (3) stratifies BMI, WC, or WHR into more than two stratifications; and (4) presents the RR, odds ratio (OR), or original data that could calculate RR values.

Studies were excluded if any of the following criteria were identified: (1) studies were case reports or case series; (2) studies were not conducted in human adults; (3) studies were conducted in population samples comprising only patients with obesity, metabolic syndrome, glaucoma, or OHT at baseline; (4) studies selected close-angle glaucoma as an outcome; and (5) studies concerned drug effects or specific conditions (e.g., eye surgery).

### 2.3. Data Extraction and Assessment of Study Quality

Data were extracted and summarized from all eligible studies by two independent reviewers (Weiming Liu and Yiyi Chen). Any disagreements were discussed by the two reviewers or resolved by adjudicating senior authors (Peirong Lu). The data included the following: name of the first author, publication year, study design, study follow-up period, country, database of the data collection from, number of participants (case/control), age, outcome definition, exposure definition, adjustment factors, and OR/RR value with a 95% CI.

Because there is no suitable standardized assessment method to assess the quality of observation studies, including cohorts, case-control, and cross-sectional design, for this meta-analysis, a quality assessment tool was designed according to MOOSE, STROBE, and references [13–16]. Two independent reviewers who were blind to each other (Weiming Liu and Yan Wu) assessed the quality scales and resolved any disagreements through discussion with senior authors (Jiawen Ling and Peirong Lu). The studies which scored eight or greater on quality scales were considered to be of a relatively high methodological quality. The detail of the items and the points of each study get are shown in Table 1.

### 2.4. Statistical Methods for the Meta-Analysis

For meta-analysis, RR with 95% CI was assessed to determine the relationship between adiposity and glaucoma incidence. Adjusted data were used to assess the relationship between adiposity and the risk of glaucoma if the adjusted and unadjusted data were reported in the articles. When the results were provided by gender, the results were summarized into a single RR with a 95% CI, using the fixed-effects method and under the assumption that OR were accurate approximations of RR [17].

Subgroup analyses were carried out according to adiposity measurement (BMI group or abdominal adiposity, including WC or WHR) and outcome definition (IOP group, or glaucoma group). In the exposure-definition subgroups, articles were divided into general adiposity (BMI) group or abdominal adiposity group (WC or WHR); the association between general or abdominal adiposity with glaucoma (including OAG or elevated IOP as outcome) was then assessed. In the outcome-definition subgroup, articles were divided into the IOP group or open-angle glaucoma group; the relationship between adiposity anthropometric stratification and IOP change or prevalence of OAG were then assessed. With the exception of the exposure-group analysis, it was preferable to use RR values on WC or WHR rather than on BMI.

Statistical heterogeneity was evaluated across studies using the *Q* test and *I*^2^ tests, where *P_Q_* < 0.1 or *I*^2^ > 50% represented significant heterogeneity across studies. Accordingly, the random-effects method was used to evaluate the potential relationship between adiposity and glaucoma for all analyses [18].

Moreover, sensitivity analyses were examined by deleting each study individually to evaluate the quality and consistency of the results. A series subgroup analysis was also conducted. Begg's test and Egger's test were used to evaluate the potential publication bias, and funnel plots were presented visually [19, 20]. All statistical analyses were carried out using the STATA software package (version 12.0; STATA Corp., College Station, TX).

## 3. Results

### 3.1. Identification and Selection of Studies

**Figure 1:**
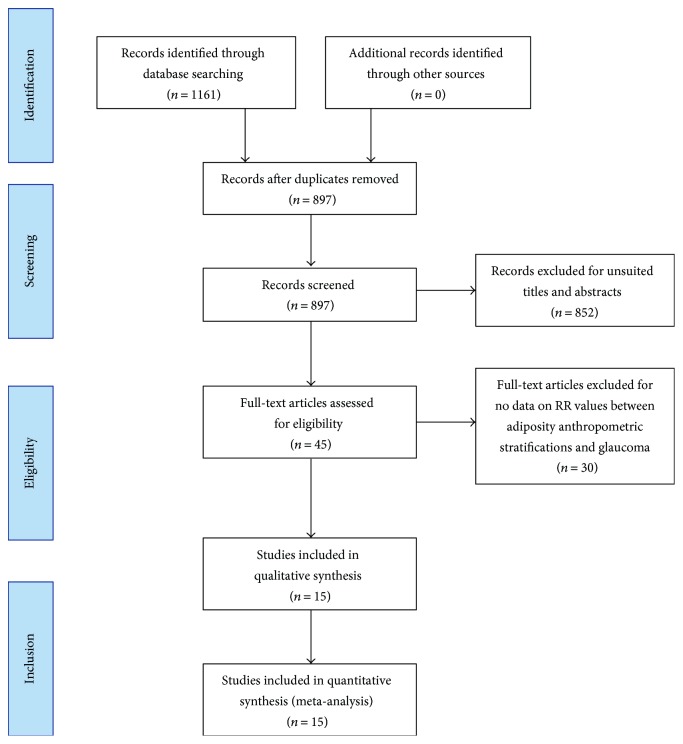
Flow diagram showing the selection process for inclusion of studies.

Initially, 1161 articles were identified, comprising 644 from PubMed and 517 from ISI Web of Science. Among these articles, there were 264 duplicates and 852 unrelated articles, which were excluded. After reading the 45 full-text articles, 30 articles were excluded because they did not provide available data on BMI, WC, or WHR stratification. Ultimately, to conduct this meta-analysis, 15 studies were identified, which had been published from 1995 to 2016. A flowchart for the literature search work and results is shown in Figure 1.

### 3.2. Study Characteristics and Quality Assessment

A total of 2,445,980 individuals from all included studies were included.  Table 2 showed the characteristics of 15 studies. In all, it was possible to identify nine cross-sectional studies [2, 8, 10, 11, 21–25], one case-control study [26], and five cohort studies [7, 27–30]. The geographic distribution of these studies was six in the America [2, 7, 22, 28–30], one in Europe [27], seven in Asia [8, 10, 11, 23–26], and one in Africa [21]. The longest study period was more than 24 years [29], and study periods were different between the included studies.

Adjusted factors differed between the included studies, such as age, sex, alcohol consumption, smoking status, physical activity, hypertension, and diabetes. The quality scale for 11 of the studies was 8 or greater, which is considered to indicate a relatively high methodological quality, and the remaining 4 studies scored less than 8 (the average scale of 15 studies was 8.3).

### 3.3. Pooled-Analysis Results

#### 3.3.1. Elevated IOP or OAG Group Analysis

**Figure 2:**
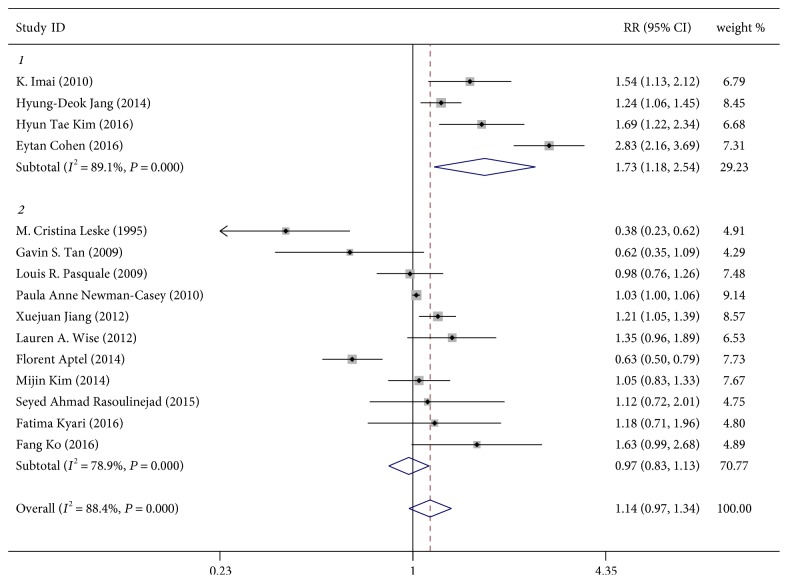
Forest plot for the association between adiposity and elevated IOP or OAG incidence. 1 = elevated IOP group; 2 = OAG group. Note: weights are from random-effects analysis.

The pooled RR for four studies using exclusively elevated IOP [8, 10, 11, 25] as an outcome was 1.73 (95% CI, 1.18–2.54, *P* = 0.005; *I*^2^ = 89.1%, *P*_heterogeneity_ < 0.001; Figure 2), whereas the RR for 11 studies using OAG [2, 7, 17–20, 22–26] as an outcome was 0.97 (95% CI, 0.83–1.13, *P* = 0.709; *I*^2^ = 78.9%, *P*_heterogeneity_ < 0.001; Figure 2).

#### 3.3.2. General or Abdominal Adiposity Group Analysis

**Figure 3:**
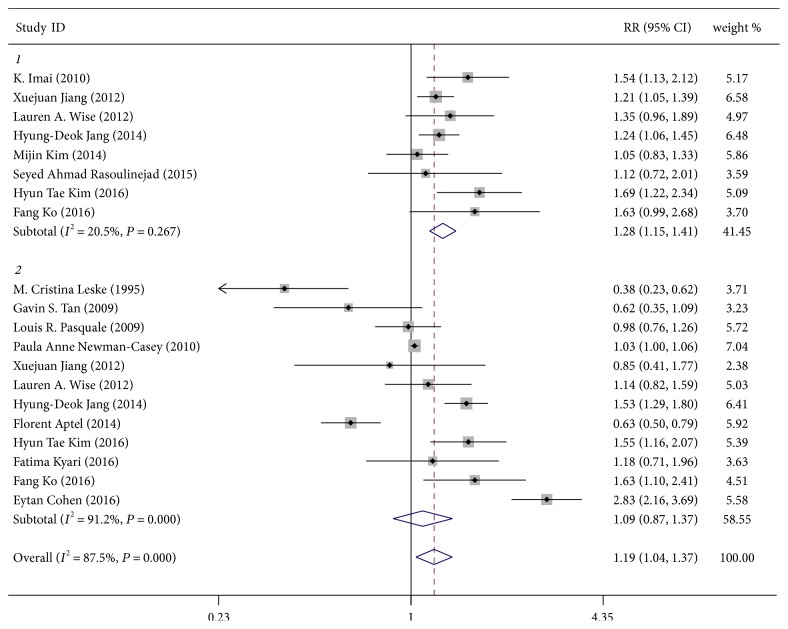
Forest plot for the association between general or abdominal adiposity and glaucoma. 1 = abdominal group (measured by waist circumference or waist-to-hip ratio); 2 = general group (measured by body mass index). Note: weights are from random-effects analysis.

The pooled RR for studies using exclusively abdominal adiposity measured by WC or WHR [7, 10, 11, 22, 24–26, 28] for exposure was 1.28 (95% CI, 1.15–1.41, *P* < 0.001; *I*^2^ = 20.5%, *P*_heterogeneity_ = 0.267; Figure 3); the heterogeneity was statistically insignificant, whereas the RR for studies using general adiposity measured by BMI [2, 7, 8, 10, 11, 21–23, 27–30] for exposure was 1.09 (95% CI, 0.87–1.37, *P* = 0.433; *I*^2^ = 91.2%, *P*_heterogeneity_ < 0.001; Figure 3).

#### 3.3.3. Subgroup Analysis

The series subgroup (Table 3) that was conducted included study design, gender, smoking, alcohol intake, physical activity, hypertension, diabetes mellitus, other metabolic syndrome components, and central corneal thickness (CCT). Results of subgroup analysis by the study design did not indicate the significant association in the cohort study group (RR, 1.00; 95% CI, 0.84–1.20; *I*^2^ = 84.1%; *P* < 0.001) or in the cross-sectional study group (RR, 1.22; 95% CI, 0.89–1.66; *I*^2^ = 88.6%; *P* < 0.001). Because there was only one case-control study, it was not included in this subgroup analysis. In the gender subgroup analysis, the pooled RR for men [2, 8, 10, 25, 29, 30] was 1.11 (95% CI, 0.77–1.60; *I*^2^ = 91.8%; *P* < 0.001), while the pooled RR for women [2, 7, 8, 10, 11, 25, 29, 30] was 1.31 (95% CI, 1.05–1.64; *I*^2^ = 80.3%; *P* < 0.001).

### 3.4. Sensitivity Analysis and Publication Bias

**Figure 4:**
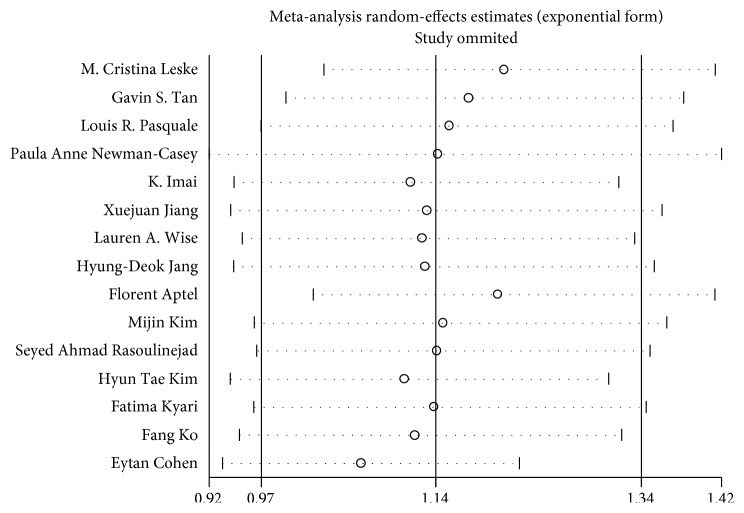
Sensitivity analysis of the association between adiposity and glaucoma.

**Figure 5:**
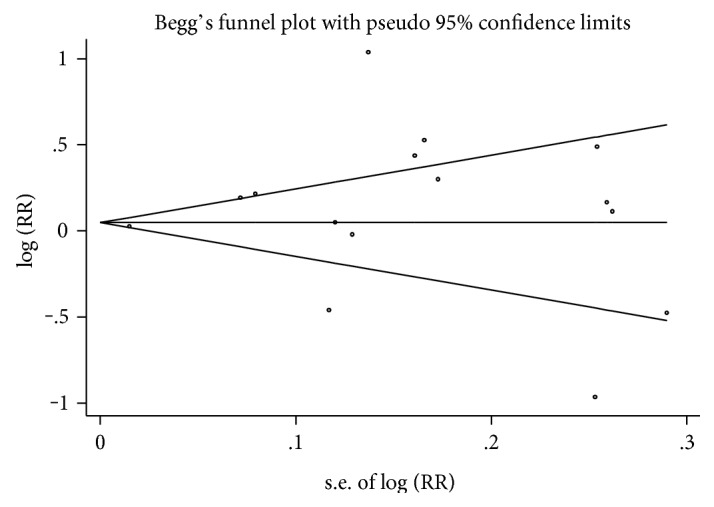
Funnel plot for studies of the association between adiposity and glaucoma.

In the analysis, a sensitivity analysis was carried out to evaluate the stability of the results by deleting one study at a time and calculating the pooled OR for the remaining studies. Apart from Leske et al. [2] (pooled RR, 1.21; 95% CI, 1.03–1.41) and Aptel et al. [27] (pooled RR, 1.20; 95% CI, 1.02–1.41), when any other study was excluded, the estimated pooled RR was similar to previously (Figure 4). Begg's funnel plot and Egger's test revealed the absence of publication bias (Figure 5). The *P* value for Begg's test was 0.843; for Egger's test, it was 0.383.

## 4. Discussion

First, a meta-analysis was conducted to summarize the evidence from all available retrospective and prospective studies in order to evaluate the association between adiposity and the risk of elevated IOP or OAG incidence. Importantly, pooled data were provided for a substantial number of cases and controls to enable better understanding of this relationship. In this study, the pooled RR value suggested that adiposity had a positive association with the risk of elevated IOP, while there is no significant association between adiposity and OAG. Second, a series of subgroup and sensitivity analyses were conducted, according to the anthropometric-parameter measurements of adiposity, as well as exposure stratification. A slight positive association between abdominal adiposity measured by WC or WHR and risk of glaucoma was found in this analysis; however, the relationship was insignificant between general adiposity measured by BMI with glaucoma incidence. Finally, it was possible to identify a relationship in the gender subgroup showing that adipose women had a higher risk of glaucoma than adipose men.

In previous studies, several theories explain the relationship between adiposity and glaucoma. One theory suggests that cerebrospinal fluid pressure (CSFP) and glaucomatous optic neuropathy may be due to either an elevated IOP, an abnormally low orbital CSFP, or higher translamina cribrosa pressure difference (TLCPD) [31, 32]. Obese patients have higher cerebrospinal fluid pressure, which may be related to a larger neuroretinal rim area equivalent to the optic nerve fibers [33, 34]. Some studies suggested that taller body height with higher CSFP and lower TLCPD resulted in a lower prevalence of OAG [32]. However, other studies suggested that a taller person with a lower BMI has a higher risk of a smaller neuroretinal rim area and a larger optic cup-to-disc area ratio. Similarly, a taller person with a lower BMI may have a higher risk for developing OAG, while a higher BMI may be a protective risk [35, 36]. These findings suggest possible biological mechanisms for the pathogenesis of OAG, but more studies are needed to research the association between adiposity and CSFP and TLCPD, which are related to the risk of glaucoma.

The second hypothesis proposes that excess orbital fat tissue may increase episcleral venous pressure and blood viscosity, with increased outflow resistance in the episcleral veins, which could cause a decreased outflow facility and an increased IOP [37]. Moreover, obese patients may have a narrower orbital optic nerve subarachnoid space, which suggests a lower orbital CSFP involved in the pathogenesis of glaucoma [38]. In addition, there is a theory that an accumulation of lipid depositions may reduce the facility of aqueous outflow and secondarily elevate IOP [39]. Higher IOP and lower anterior chamber depth (ACD) would be significantly related to obesity [40].

Another possible mechanism supporting the association between adiposity and IOP is that hyperleptinemia, which accompanies obesity, may result in increased oxidative stress [41]. Compared to healthy subjects, the trabecular meshwork of obese patients with OAG has higher oxidative damage [42]. Physical fitness has been shown to decrease IOP temporarily. Exercise has an effect on decreasing IOP because of lower norepinephrine concentrations, increased colloid osmotic pressure, coaction of nitric oxide, endothelin, and a *β*2-adrenergic receptor gene polymorphism [43, 44]. Almost all obese patients with glaucoma and elevated IOP possibly are related to this mechanism because most obese patients undertake little exercise.

Furthermore, another study has suggested that the Valsalva maneuver, or breath-holding and thorax compression, may cause transitory elevation in IOP when obese patients were measured using the Goldmann tonometry [45, 46]. Although the aforementioned mechanisms may be the cause of increased IOP elevation in obese patients, further studies are still required to better understand the mechanisms.

It was also found that abdominal adiposity measured by WC or WHR had a slight positive association with glaucoma and elevated IOP. However, these results have not been found in general adiposity measured by the BMI subgroup. This finding perhaps resulted from the use of different anthropometric methods. BMI was calculated as body weight/height^2^ (kg/m^2^) and categorized as follows: normal weight (BMI = 18.5–24.9), overweight (BMI = 25.0–29.9), and obese (BMI ≥ 30) [47]. Ethnic-specific waist circumference cut-offs have been incorporated into the definition. In Asian people, the relevant WC is above 90 cm in men and 80 cm in women, and in European people, it rises to above 94 cm in men and 80 cm in women [48]. Although BMI is used widely, it cannot measure adiposity parameters. Abdominal obesity plays an important role in the metabolic syndrome, and WC rather than BMI is recommended as the measurement [49].

WHR is a relatively new anthropometric index that is normalized by body size, which was proposed in the 1990s [50]. WHR is demonstrated as a better predictor of disease or mortality risk than BMI [51], and it may be more related to age-related diseases, since muscle loss and changes in regional adipose tissue distribution are common with aging [52]. Among several anthropometric parameters, greater WC or WHR was correlated with higher IOP, but this association was of little clinical significance. WC or WHR is recognized as a good predictor of abdominal adiposity, while BMI does not reflect actual body condition. Moreover, Kim et al. also found that other adiposity parameters were related to elevated IOP, such as a higher fat ratio and lower lean body mass (muscle mass) and bone-mineral content ratio [11]. More studies are needed to confirm which is the best measurement tool for obese patients.

Previous studies found that overall prevalence of overweight/obesity was higher in females than in males, and the prevalence of central obesity was also higher in females than in males [53]. Intraocular pressure changes may be linked to metabolic syndrome in postmenopausal women, but not in premenopausal women [54, 55]. Because men and women have such different body compositions, in this study, the data were stratified according to sex. The results revealed that the anthropometric parameters of adiposity were associated with IOP, especially among women. Estrogen may also play an important role, and ESR2 gene polymorphism is considered to be associated with elevated IOP in female patients with OAG [56]. Estrogen can regulate smooth muscle tone and vascular resistance and, as a result, augments the activity of endothelial-based nitric oxide synthase and has a hypertensive effect by influencing aqueous production and outflow systems [57]. Gimeno and Klaman [58] suggested that adipose tissue may serve as an endocrine organ and secrete other paracrine factors that could also influence retinal ganglion cell health. Nevertheless, the underlying mechanisms between obesity, sex, and OAG are unclear.

However, several potential limitations should be taken into account in the interpretation of these findings. First, the main limitation is that only five studies were prospective and the others were retrospective. There was no standard method to assess the quality of the included studies, and substantial heterogeneity was found in the methods and quality of the original studies. Significant variability was found in terms of the study design, exclusion criteria, inclusion criteria, definition of exposure and outcomes, method of diagnosis of glaucoma, IOP measurement, and the difference between anthropometric-parameter measurements for adiposity. For instance, abdominal adiposity was measured by WC or WHR, while general adiposity was measured by BMI. Begg's funnel plot showed a little asymmetry because of the heterogeneity between studies. All the aforementioned factors could be the source of the high heterogeneity between the studies.

Second, the heterogeneity of uncontrolled or residual confounding from different original studies may influence the present study's results. The more components of metabolic syndrome a person has, the higher the risk of OAG. Metabolic-syndrome components including obesity, hypertension, diabetes mellitus, and hyperlipidemia may play a role in the pathogenesis of OAG [24, 25, 30]. Not only for hypertension [59] and diabetes mellitus, another meta-analysis also found that obstructive sleep apnea syndrome has a relationship with elevated IOP and the relevance of glaucoma [15]. Moreover, obese patients almost always combined the above systematic factors with bad habits such as alcohol consumption, cigarette smoking, and little activity [60]. Despite using the random-effects model to summarize the pooled estimate data in order to minimize the effects of heterogeneous variability between studies, the residual or unmeasured confounding factors may affect the results of the present study.

Third, potential selection bias is likely to exist. Different studies with different cases and control participants resulted in selection bias because different ophthalmologists would not have used exactly the same criteria. In addition, studies were limited to the English language, which may mean that data were missed from important studies published in other languages. Finally, despite the comprehensive search of all relative articles, many articles did not meet the inclusion criteria. Publication bias was still inevitable because research with null results was not reported.

## 5. Conclusions

In the meta-analysis, it was found that adiposity has a higher risk of elevated IOP and abdominal adiposity has a positive association with glaucoma, especially in female patients. Future research should focus on longitudinal cohort studies with objective measurements for adiposity and should consider the mechanisms for obesity and the risk of glaucoma. Understanding the systematic factors associated with elevated IOP and OAG progression will help clinicians in screening and monitoring patients in the early stages.

## Figures and Tables

**Table 1:** Quality assessment of each study.

Scale items	First author														
	M. Cristina Leske	Gavin S. Tan	Louis R. Pasquale	Paula Anne	K. Imai	Xuejuan Jiang	Lauren A. Wise	Hyung-Deok Jang	Florent Aptel	Mijin Kim	Seyed Ahmad	Hyun Tae Kim	Fatima Kyari	Fang Ko	Eytan Cohen
(1) Whether the study was cohort study	−	−	+	+	−	+	+	−	+	−	−	−	−	−	−
(2) Whether the study listed the inclusion and exclusion criteria	+	+	+	+	+	+	+	+	+	+	+	+	+	+	+
(3) Whether the study described the setting, locations, and relevant dates, including periods of recruitment, exposure, follow-up, and data collection	+	+	+	+	+	+	+	+	+	+	+	+	+	+	+
(4) Whether the study clearly define all outcomes, exposures, and potential confounders	+	+	−	−	+	+	+	+	−	+	+	+	+	+	+
(5) Whether the diagnosis of glaucoma was made by ophthalmologist (not based on self-reporting) or the IOP was measured by Goldmann applanation tonometer	+	+	+	+	−	+	+	+	+	+	+	+	+	+	+
(6) Whether the BMI/WC/WHR was measured by physician using standard method (not based on self-reporting)	+	+	−	−	+	+	−	+	−	+	+	+	+	+	−
(7) Whether the study described the characteristics of the study population	+	+	+	+	+	+	+	+	+	+	+	+	+	+	+
(8) Whether the study stratified BMI or WC or WHR into more than two stratifications	+	+	+	−	−	+	+	+	−	−	−	+	+	−	+
(9) Whether the study adjusted the confounding factors	+	+	+	+	+	+	+	+	+	+	+	+	+	+	+
(10) Whether the study discussed the limitation and potential bias of the study	+	+	+	+	+	+	+	+	+	+	−	+	+	+	+
Total	9	9	8	7	7	10	9	9	7	8	7	9	9	8	8

One point was allocated for above items, each item scoring 0 or 1, 1 being better. The studies with 8 scales or greater are considered the relatively high methodological quality.

**Table 2:** Characteristics of eligible studies.

First author (publication year)	Country	Study design	Database of data collection from or study follow-up period	Participants (case/control)	Age	Exposure assessment	Outcomes	Adjusted factors
M. Cristina Leske (1995)	USA	Cross-sectional	Data collection in 1995, the Barbados Eye Study	302/3821	40–84	BMI (high, medium, low)	OAG	1–3, 10
Gavin S. Tan (2009)	Singapore	Cross-sectional	Data collection in 2009, the Singapore Malay Eye Study	102/3146	40–80	BMI (>25)	OAG	1, 2, 4, 8, 9, 11
Louis R. Pasquale (2009)	USA	Cohort study	Follow-up period from 1980 to 2004 for women; follow-up period from 1986 to 2004 for men; followed every 2 years, NHS and HPFS	Women 642/78,135 men 338/41,014	>40	BMI (<22, 22-23.9, 24-25.9, 26-27.9, 28-29.9, >30)	OAG	1, 3–6, 13
Paula Anne Newman-Casey(2010)	USA	Cohort study	Follow-up from 2001 to 2007	55,090/2,127,225	40–87	BMI	OAG	1, 2, 7, 8, 10–13, 16, 17
K. Imai (2010)	Japan	Cross-sectional	Data collection in 2009, Health Checkup Program(2004–2008)	14,003 participants	18–83	WC	Elevated IOP	No
Xuejuan Jiang (2012)	USA	Cohort study	4 years from baseline (2000–2003) to follow-up (2004–2008), LALES	87/3685	>40	WHR (per 0.05 higher); BMI (<25, 25–30, >30)	OAG	1, 9, 14, 15
Lauren A. Wise (2012)	USA	Cohort study	Follow-up from 1995 to 2007; followed every 2 years, BWHS	366/32,204	21–69	WHR (<0.72, 0.72–0.77, 0.78–0.84, >0.85); BMI (<25, 25–29, 30–34, >35)	OAG	1, 4–7, 11
Hyung-Deok Jang (2014)	Korea	Cross-sectional	Data collection from KNHANES 2008–2010 database	15,271 participants	>19	WC (>90 for men, >85 for women); BMI(>25)	Elevated IOP	1, 4–8
Florent Aptel (2014)	France	Cohort study	Follow up from 2009 to 2012	330/9250	>50	BMI (>30)	OAG	1, 2, 7, 12
Mijin Kim (2014)	Korea	Cross-sectional	Data collection from 2010 to 2011	300/17,940	>40	WC (>90 for men, >80 for women)	OAG	12
Seyed Ahmad Rasoulinejad (2015)	Iran	Case-control	Data collection in 2015	100/100	>18	WC (>102 for men, >88 for women)	OAG	12
Hyun Tae Kim (2016)	Korea	Cross-sectional	Data collection from 2010-2011database	5008 participants	>19	WC (<72, 72–77, 78–84, >84 for women); BMI (<22, 22-23, 24-25, >25)	Elevated IOP	1, 4–8
Fatima Kyari (2016)	Nigeria	Cross-sectional	Data collection from 2005 to 2007, Nigeria National Blindness	462/12,738	>40	BMI (<18.5, 18.5–24.9, 25.0–29.9, >30)	OAG	1, 2, 7, 14, 15
Fang Ko (2016)	USA	Cross-sectional	Data collection from NHANES (2005–2008 cycles)	172/5574	>40	WC (>102 for men, >88 for women); BMI (>30)	OAG	1, 2, 13
Eytan Cohen (2016)	Israel	Cross-sectional	Data collection from 2000–2013 health database	18,575 participants	20–80	BMI (<25, 25–29.9, 30–35, >35)	Elevated IOP	1, 7, 8

BMI: body mass index (kg/m^2^); WC: waist circumference (cm); WHR: waist-to-hip ratio; OAG: open-angle glaucoma; IOP: intraocular pressure; NHS: the Nurses' Health Study; HPFS: Health Professionals Follow-Up Study; LALES: the Los Angeles Latino Eye Study; BWHS: the Black Women's Health Study; KNHANES: the Korea National Health and Nutrition Examination Survey; NHANES: National Health and Nutrition Examination Survey; adjusted factors: 1 = age; 2 = gender; 3 = glaucoma family history; 4 = smoking; 5 = alcohol intake; 6 = physical activity; 7 = hypertension; 8 = diabetes mellitus; 9 = CCT; 10 = cataract history; 11 = education; 12 = other metabolic syndrome components; 13 = race; 14 = IOP; 15 = AL; 16 = sleep apnea; 17 = migraine headache.

**Table 3:** Results of subgroup analysis between adiposity and glaucoma with pooled RR.

	Subgroups	Number of studies	RR (95% CI)	*I^2^*, %	Heterogeneity *P* value
Study design	Cohort	5	1.00 (0.84–1.20)	84.10%	<0.001
	Cross-sectional	9	1.22 (0.89–1.66)	88.60%	<0.001
Gender	Male	6	1.11 (0.77–1.60)	91.80%	<0.001
	Female	8	1.31 (1.05–1.64)	80.30%	<0.001
Smoking	Yes	5	1.18 (0.93–1.48)	68.20%	0.014
	No	10	1.13 (0.90–1.41)	91.20%	<0.001
Alcohol intake	Yes	4	1.26 (1.04–1.54)	57.60%	0.069
	No	11	1.09 (0.87–1.35)	90.50%	<0.001
Physical activity	Yes	4	1.26 (1.04–1.54)	57.60%	0.069
	No	11	1.09 (0.87–1.35)	90.50%	<0.001
Hypertension	Yes	7	1.28 (0.98–1.69)	93.20%	<0.001
	No	8	1.02 (0.80–1.29)	77.50%	<0.001
Diabetes mellitus	Yes	5	1.35 (0.96–1.91)	94.30%	<0.001
	No	10	1.03 (0.83–1.29)	82.20%	<0.001
Other metabolic syndrome components	Yes	4	0.92 (0.72–1.18)	82.60%	0.001
	No	11	1.23 (0.98–1.56)	86.30%	<0.001
CCT	Yes	2	0.92 (0.48–1.75)	80.10%	0.025
	No	13	1.17 (0.97–1.41)	89.40%	<0.001

RR: relative risk; CI: confidence interval; CCT: central corneal thickness.
